# 2483 The Empower Lab: An innovative model for research experience and training for undergraduate, graduate, and medical students

**DOI:** 10.1017/cts.2018.133

**Published:** 2018-11-21

**Authors:** Veronica Ades, Anna Chessky, Ydelsie Vasquez, Emily Rabinowitz

**Affiliations:** New York University General Clinical Research Center

## Abstract

OBJECTIVES/SPECIFIC AIMS: The Empower Lab was established in 2015 with the goal of providing students with hands-on research experience in sexual and gender-based violence and health. METHODS/STUDY POPULATION: The Empower Lab consists of 10–12 undergraduate, graduate, and medical students at a time. Students undergo a rigorous application process, and agree to volunteer 8 hours per week for at least 1 year. Students are assigned to teams, and learn research skills such as literature searches, systematic reviews, research question generation, study design, IRB procedures, database creation and management, data collection and analysis, oral and poster presentation, manuscript preparation, team collaboration and communication, advocacy, and leadership. Students start as research assistants, and can be promoted to team leader, and associate director of research. Students mentor and teach each other, and are supervised by the principal investigator (PI). A survey skill self-assessment is administered to lab members on entry to the lab, every 4–6 months, and upon exit. RESULTS/ANTICIPATED RESULTS: In total, 20 students have participated in the lab to date, and 12 are currently enrolled. Eighty percent of the lab members are women. The students are 45% undergraduates, 15% graduate (nursing, social work, public health), 20% medical students, and 10% not currently enrolled in school (gap year). Twenty students completed entry surveys, 11 students have completed interim surveys, and 5 students have completed exit surveys. Examination of current surveys indicates that students are gaining skills throughout the lab experience. Free-text feedback provided further insight. Currently, the lab has 5 IRB-approved studies actively recruiting participants, 4 manuscripts being written, and 3 studies in the development phase. Students have presented at three local and 2 national meetings to date. Changes have been made to the lab structure over time in order to provide clear expectations and feedback, and strengthen student performance. DISCUSSION/SIGNIFICANCE OF IMPACT: The Empower Lab is an innovative public health lab that provides opportunities for real-world research experience for students. The teamwork, collaboration, and structure of the lab permit mentoring, support, and teaching from peers, as well as from the PI. The Lab increases the PI’s productivity. Students are encouraged to develop and implement their own research ideas, further encouraging independence and initiative. Although the number of surveys is limited to date, they indicate improvement in skills and confidence among lab members. The predominance of women in the lab suggests that this is a strong model for recruitment and retention of women in STEM.

